# Magnesium Sulfate as a Multimodal Anesthetic Adjuvant in Brain Tumor Surgery: A Systematic Review and Meta-Analysis of Hemodynamic, Analgesic, and Biomarker Outcomes

**DOI:** 10.3390/jcm15124636

**Published:** 2026-06-15

**Authors:** Khairunnisai Tarimah, Iwan Fu’adi, Elvan Wiyarta, Lisda Amalia, Tatang Bisri, Dewi Yulianti Bisri

**Affiliations:** 1 Doctoral Study Program in Medical Science, Faculty of Medicine, Universitas Padjadjaran, Ir. Soekarno Street Km. 21, Jatinangor, Sumedang 45363, Indonesia; khairunnisai22001@mail.unpad.ac.id; 2 Department of Anesthesia and Intensive Care, RSUD H.Muh Roeslan Mataram City, Faculty of Medicine, Universitas Islam Al Azhar Mataram, Lombok 83237, Indonesia; 3 Department of Anesthesia and Intensive Care, Division of Neuroanesthesia and Critical Care, Faculty of Medicine, Universitas Padjadjaran, Ir. Soekarno Street Km. 21, Jatinangor, Sumedang 45363, Indonesia; iwan.fuadi@unpad.ac.id; 4 Department of Neurology, Faculty of Medicine, Universitas Indonesia, Jawa Barat 16424, Indonesia; elvan.wiyarta@ui.ac.id; 5 Department of Neurology, Faculty of Medicine, Universitas Padjadjaran, Ir. Soekarno Street Km. 21, Jatinangor, Sumedang 45363, Indonesia; lisda@unpad.ac.id; 6 Faculty of Medicine, Universitas Jendral Achmad Yani, Bandung 40525, Indonesia; tatang.bisri@yahoo.co.id

**Keywords:** magnesium sulfate, craniotomy, hemodynamics, biomarkers, S100B protein

## Abstract

**Background/Objectives:** Strict hemodynamic stability is critical during supratentorial craniotomy. This systematic review and meta-analysis aimed to evaluate the efficacy of magnesium sulfate (MgSO_4_) as a multimodal adjuvant on intraoperative hemodynamics, opioid consumption, and biomarker outcomes in this setting. **Methods:** We systematically searched PubMed, Scopus, EBSCO, and the Cochrane Library for randomized controlled trials (RCTs) comparing perioperative MgSO_4_ administration to placebo or standard care in adult patients undergoing elective supratentorial craniotomy. **Results:** Meta-analysis of nine included RCTs using a random-effects model demonstrated that MgSO_4_ significantly reduced intraoperative mean arterial pressure (mean difference [MD]: −4.65 mmHg; 95% confidence interval [CI]: −7.76 to −1.55; *p* = 0.0033; I^2^ = 73.6%). Furthermore, MgSO_4_ administration significantly lowered postoperative serum S100B levels (standardized MD [SMD]: −0.81; 95% CI: −1.24 to −0.38; *p* = 0.0002, I^2^ = 0.0%), indicating mitigated cellular neural damage, and decreased perioperative fentanyl consumption (standardized MD: −1.01; 95% CI: −1.45 to −0.57; *p* < 0.0001; I^2^ = 0.0%). Intraoperative blood loss volume did not differ significantly between groups (MD: −85.03 mL; 95% CI: −331.42 to 161.37; *p* = 0.4952; I^2^ = 92.5%). **Conclusions:** MgSO_4_ is a safe and effective multimodal adjuvant for supratentorial craniotomy, providing significant hemodynamic stability, opioid-sparing effects, and preliminary biochemical evidence suggestive of neuroprotection without compromising intraoperative hemostasis.

## 1. Introduction

Strict hemodynamic stability is critical during supratentorial craniotomy to prevent dangerous surges in intracranial pressure (ICP) and intraoperative brain swelling. Acute hypertensive episodes are frequently triggered by noxious stimuli such as skull pin insertion and laryngoscopy, which can rapidly increase cerebral blood volume in patients with already compromised intracranial compliance [1]. The Anesthesia Patient Safety Foundation (APSF) identifies perioperative hemodynamic instability as a primary preventable cause of organ injury, emphasizing the need for tight control [2,3]. While high-dose opioids are traditionally used to blunt these sympathetic responses, they are associated with dose-dependent adverse effects including postoperative nausea and vomiting (PONV), and respiratory depression [4,5]. These complications are particularly hazardous in neurosurgery because vomiting and hypercapnia can further elevate ICP and obscure neurological assessment, highlighting the urgent need for effective multimodal adjuvants that minimize opioid requirements [4,6].

Magnesium sulfate (MgSO_4_) has gained attention as a multimodal agent due to its dual mechanism as a physiological calcium channel blocker and a non-competitive N-methyl-D-aspartate (NMDA) receptor antagonist [7,8]. By blocking the voltage-dependent NMDA pore, magnesium inhibits excessive calcium influx and glutamate release, which are fundamental drivers of central sensitization and neuronal excitotoxicity [9,10]. Clinical evidence from spinal and vascular surgery demonstrates that intravenous magnesium effectively reduces postoperative pain scores and opioid consumption by approximately 30% while stabilizing hemodynamics without increasing the risk of hypotension or acute kidney injury [11,12]. Furthermore, in vitro studies on human brain organoids suggest that magnesium upregulates GABA receptors and Brain-Derived Neurotrophic Factor (BDNF), implying a potential neuroprotective role against surgical stress and ischemia [8,13].

Despite these theoretical benefits, current evidence regarding the efficacy of MgSO_4_ in supratentorial craniotomy remains fragmented and inconclusive. Previous systematic reviews have largely focused on spinal surgery or regional anesthesia and often fail to address specific neurosurgical outcomes such as neuroprotection [7]. There is a notable lack of synthesized data evaluating the impact of intraoperative magnesium on serum S100B levels, a specific biomarker for blood–brain barrier disruption and glial injury [11]. Additionally, it remains unclear whether the efficacy of magnesium varies between total intravenous anesthesia (TIVA) and inhalational maintenance, as prior analyses have not systematically stratified these subgroups. Therefore, this systematic review and meta-analysis aim to evaluate the efficacy of magnesium sulfate in supratentorial craniotomy, with a specific focus on hemodynamic stability, opioid consumption, and preliminary biochemical markers suggestive of neuroprotection.

## 2. Materials and Methods

### 2.1. Search Strategy and Data Sources

This systematic review and meta-analysis was conducted in strict adherence to the Preferred Reporting Items for Systematic Reviews and Meta-Analyses (PRISMA), and the protocol was prospectively registered in the PROSPERO database (CRD420251238665). A comprehensive systematic search was performed across major electronic databases, including PubMed, Scopus, EBSCO, and The Cochrane Library, covering the period from inception to 15 January 2026. The search strategy employed a combination of Medical Subject Headings (MeSH) terms and free-text keywords related to the intervention and population, specifically “Magnesium Sulfate,” “MgSO_4_,” “Craniotomy,” “Supratentorial neoplasm,” “Brain Tumor,” “Neurosurgery,” and “Anesthesia,” without applying any language restrictions. The specific Boolean search strings used for each database and the corresponding number of records retrieved are detailed in Supplementary Table S1. To ensure the completeness of the literature retrieval, we also manually screened the reference lists of included studies and relevant review articles to identify potential additional trials that might have been missed by the electronic search.

### 2.2. Eligibility Criteria

Studies were strictly selected based on the PICOS framework, focusing exclusively on Randomized Controlled Trials (RCTs) that enrolled adult patients aged 18 years or older undergoing elective supratentorial craniotomy, such as for tumor resection or epilepsy surgery, under general anesthesia. The intervention of interest was the perioperative administration of Magnesium Sulfate (MgSO_4_) via intravenous infusion or as a regional adjuvant, compared against a placebo or standard care control group. We specifically included studies that reported quantitative data on intraoperative hemodynamic stability as the primary outcome, as well as secondary outcomes including biochemical markers suggestive of neuroprotection (serum S100B), intraoperative blood loss, perioperative opioid consumption, and postoperative pain scores. Observational studies, case reports, pediatric populations, and trials with insufficient data for extraction were excluded from this review.

### 2.3. Data Extraction and Quality Assessment

Two independent reviewers screened titles, abstracts, and full texts to determine eligibility and subsequently performed data extraction using a standardized pilot-tested form. This process captured key details including study characteristics, patient demographics, magnesium dosing regimens (which were standardized to mg/kg for consistency across studies), specific outcome measures, and anesthesia maintenance protocols (specifically distinguishing between total intravenous anesthesia and inhalational techniques). The methodological quality of the included RCTs was rigorously assessed using the Cochrane Risk of Bias 2.0 tool (RoB 2), evaluating domains such as the randomization process, deviations from intended interventions, missing outcome data, measurement of outcomes, and selection of reported results. Detailed justifications for each domain-level judgment, particularly for downgraded studies, are provided in a Supplementary Table S2. To conclude the assessment, the certainty of the evidence for each outcome was graded according to the Grading of Recommendations Assessment, Development, and Evaluation (GRADE) approach, which categorizes evidence quality based on risk of bias, inconsistency, indirectness, imprecision, and publication bias. Specific reasons for downgrading evidence certainty, such as small-study effects or substantial heterogeneity, are explicitly outlined in the results.

### 2.4. Statistical Analysis

Meta-analysis was conducted using RevMan 5 with a random-effects model (Restricted Maximum Likelihood estimator) for all analyses to account for the clinical and methodological diversity among the included trials. For continuous outcomes reported in identical units, specifically Mean Arterial Pressure (MAP) and intraoperative blood loss volume, the Mean Difference (MD) was calculated with 95% Confidence Intervals (CIs). Conversely, the Standardized Mean Difference (SMD) was utilized for cumulative opioid consumption to accommodate variations in reporting metrics (e.g., total cumulative dose versus infusion rates) across studies, and for serum S100B levels to account for potential differences in laboratory biomarker kits. Statistical heterogeneity was quantified using the I^2^ statistic, where an I^2^ value greater than 50% indicated substantial heterogeneity. Although the number of included studies per outcome was limited (k < 10), a funnel plot was generated for the primary outcome (MAP) to visually assess potential publication bias, which was interpreted cautiously in accordance with the Cochrane Handbook guidelines. Furthermore, to explore and address substantial heterogeneity, prespecified subgroup analyses were conducted by stratifying studies based on anesthetic maintenance techniques (TIVA versus Inhalational). Exploratory leave-one-out sensitivity analyses and targeted exclusions (e.g., isolating strict intravenous administration from regional scalp block protocols) were also performed to confirm the robustness and methodological consistency of the pooled findings.

## 3. Results

### 3.1. Study Selection and Baseline Characteristics

**Figure 1:**
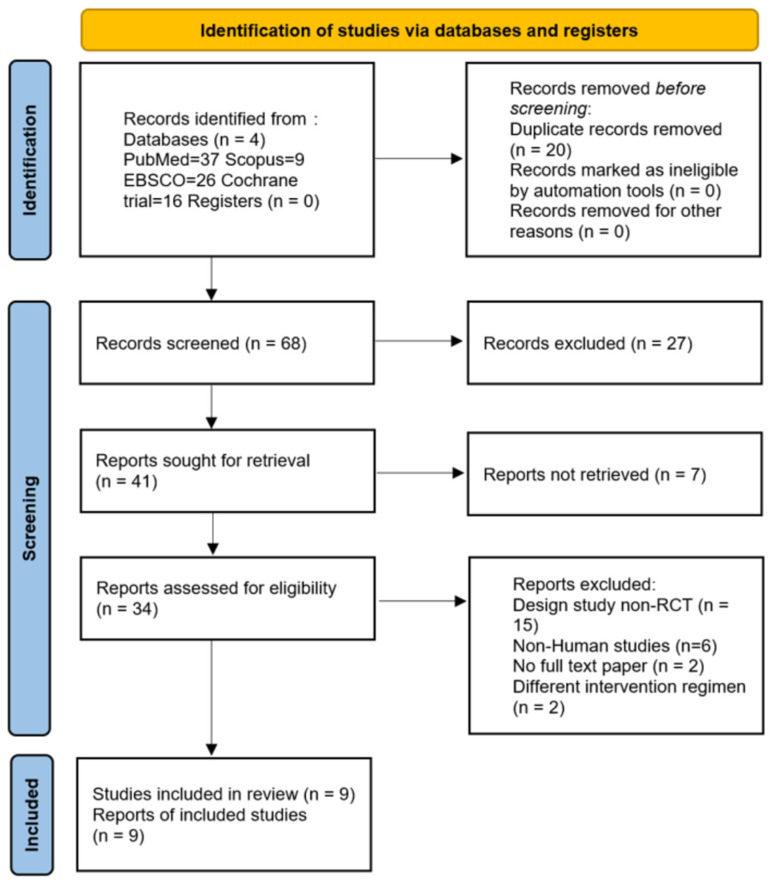
PRISMA Flowchart.

The study selection process involved a comprehensive search across four major databases, including PubMed, Scopus, EBSCO, and Cochrane trials. As shown in Figure 1, this systematic approach ensured that all relevant records were identified before undergoing a rigorous screening process to remove duplicates and ineligible papers.

A total of nine randomized controlled trials were identified and included in the final quantitative synthesis. These studies were conducted across diverse geographical settings, including Iran, Thailand, Egypt, India, and Spain, ensuring a relatively broad representation of clinical practices. The included trials exclusively recruited adult patients undergoing supratentorial neurosurgical procedures, such as tumor resection and epilepsy surgery. The intervention protocols predominantly involved the systemic intravenous administration of Magnesium Sulfate (MgSO_4_) with varying loading and maintenance regimens (which were standardized to mg/kg for all analyses to ensure consistency), while one study utilized MgSO_4_ as a regional adjuvant in a scalp block [14]. Detailed demographic data, anesthesia maintenance protocols (TIVA versus Inhalational), and specific dosage regimens for each trial are summarized in Table 1.

### 3.2. Risk of Bias Assessment and Certainty of Evidence

**Figure 2:**
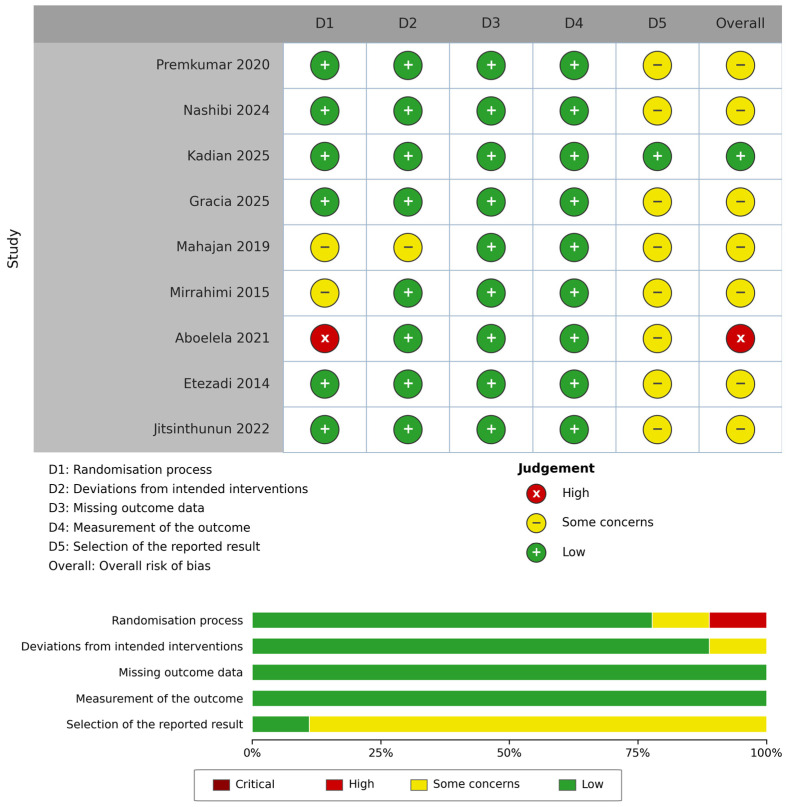
RoB 2.0 [14,15,16,17,18,19,20,21,22].

The methodological quality of the included randomized controlled trials was rigorously assessed using the Cochrane Risk of Bias 2.0 tool (RoB 2.0). Contrary to broad summary statements, the assessment revealed that the risk of bias varied across domains. As illustrated in Figure 2, several studies were downgraded from ‘Low Risk’ to ‘Some Concerns’ or ‘High Risk’ in specific domains, particularly concerning unclear allocation concealment, small sample sizes, and the lack of pre-registered study protocols. Detailed domain-level justifications for these downgraded judgments are provided in Supplementary Table S2. Furthermore, the certainty of evidence for each outcome was graded according to the GRADE approach, with levels downgraded to account for the identified risk of bias, inconsistency in pooled results, and imprecision resulting from limited sample sizes.

### 3.3. Primary Outcome Analysis: Intraoperative Hemodynamics

**Figure 3:**
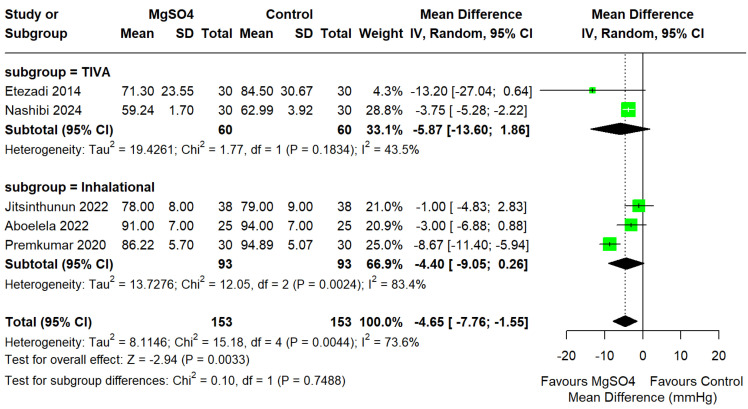
Forest Plot of Intraoperative Mean Arterial Pressure (MAP) comparing Magnesium Sulfate versus Control [15,18,19,20,21].

Randomized controlled trials provided sufficient quantitative data (mean and standard deviation) to assess intraoperative Mean Arterial Pressure (MAP). Given the variability in clinical protocols across the included studies, a random-effects model was employed to determine the pooled effect size. The quantitative synthesis comparing the hemodynamic profile between the Magnesium Sulfate (MgSO_4_) group and the Control group, stratified by anesthetic maintenance technique (TIVA versus inhalational anesthesia), is presented in Figure 3. The meta-analysis utilizing a random-effects model demonstrates that the administration of Magnesium Sulfate (MgSO_4_) is associated with a statistically significant reduction in intraoperative Mean Arterial Pressure (MAP) compared to the control group, yielding a pooled mean difference of −4.65 mmHg (95% CI: −7.76 to −1.55; *p* = 0.0033).

Although statistically significant, the clinical meaningfulness of a 4.65 mmHg reduction in MAP remains debatable, as it may not directly translate into robust postoperative clinical benefits, such as reduced cerebral edema or shorter hospital stays. Furthermore, substantial heterogeneity was detected among the included studies (I^2^ = 73.6%; *p* = 0.0044). To address this clinical diversity, an exploratory subgroup analysis based on anesthetic technique was performed. The analysis revealed that heterogeneity remained high within the inhalational subgroup (I^2^ = 83.4%) but was notably lower in the TIVA subgroup (I^2^ = 43.5%). This suggests that while anesthetic technique accounts for a portion of the variability, other factors such as disparities in magnesium dosing protocols, intravenous versus regional administration routes, and surgical indications also contribute significantly. Consequently, while the aggregate data suggests a hemodynamic stabilizing effect of MgSO_4_, these findings should be interpreted with caution and contextualized within the specific anesthetic regimens utilized.

### 3.4. Secondary Outcome Analysis

#### 3.4.1. Neuroprotection (S100B Biomarker)

**Figure 4:**
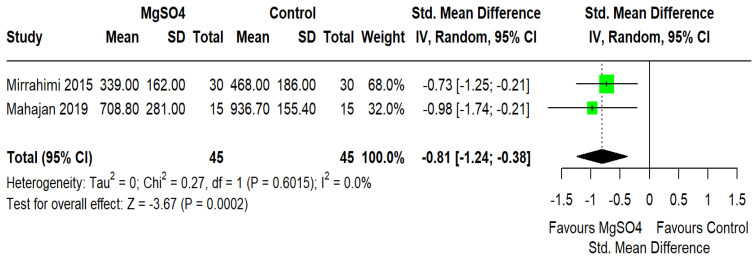
Forest Plot of Postoperative S100B Biomarker Levels [16,17].

To evaluate the potential neuroprotective effects of the intervention, postoperative serum S100B protein levels were analyzed as a biochemical surrogate for neuronal injury and blood–brain barrier disruption. Quantitative data suitable for meta-analysis were extracted from two randomized controlled trials (*n* = 90). Although both included studies reported S100B concentrations in identical units (pg/mL), the Standardized Mean Difference (SMD) was selected as the appropriate effect measure to account for potential variations in biomarker assay kits across different study centers. A random-effects model was applied to synthesize the findings, and the resulting forest plot comparing the Magnesium Sulfate and control groups is presented in Figure 4.

The pooled analysis demonstrated a statistically significant reduction in postoperative S100B levels in the Magnesium Sulfate group compared to the control group, yielding a pooled standardized mean difference of −0.81 (95% CI: −1.24 to −0.38; *p* = 0.0002). This reduction indicates a potential mitigation of cellular neural damage following the surgical procedure, though it must be emphasized that biomarker reduction alone is insufficient to conclude clinically meaningful neuroprotection without robust patient-centered neurological outcomes. Notably, the analysis revealed zero heterogeneity across the included studies (I^2^ = 0.0%; *p* = 0.6015), suggesting a consistent biochemical response across the observed trials. Given that these studies employed a Total Intravenous Anesthesia (TIVA) protocol, these findings specifically substantiate the efficacy of intraoperative magnesium administration in attenuating neuronal stress within this anesthetic context.

#### 3.4.2. Intraoperative Blood Loss Volume

**Figure 5:**
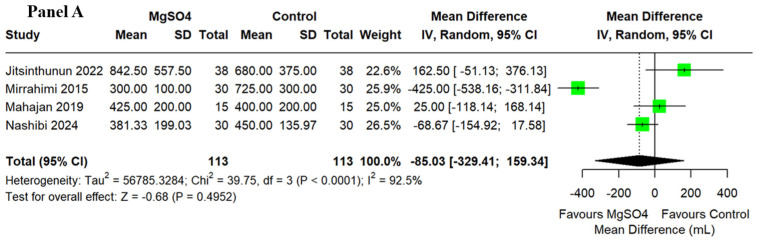
Forest plots of Intraoperative blood loss volume (mL) [16,17,19,21].

Intraoperative blood loss volume was assessed as a key secondary endpoint to determine the hemostatic influence of the intervention, with data extractable from four randomized controlled trials (n = 226). Given that all included studies utilized a consistent unit of measurement (milliliters), the Mean Difference (MD) was selected as the appropriate effect measure rather than the Standardized Mean Difference (SMD), thereby ensuring a direct comparison of the absolute volume reduced. A random-effects model was employed to synthesize the data, accounting for the anticipated methodological and clinical diversity across the different surgical centers. The resulting forest plot describing the difference in blood loss between the Magnesium Sulfate and control groups is presented in Figure 5.

The quantitative synthesis revealed that patients receiving Magnesium Sulfate experienced a lower mean blood loss compared to the control group, with a pooled mean difference of −85.03 mL. However, this reduction did not reach statistical significance (*p* = 0.4952), as the 95% confidence interval (−329.41 to 159.34) spans the line of no effect. Furthermore, a substantial degree of heterogeneity was observed (I^2^ = 92.5%; *p* < 0.0001), indicating high variability in surgical complexity or measurement techniques among the studies. Consequently, while a trend toward reduced bleeding is observed, the high heterogeneity and wide confidence intervals suggest that the evidence is currently insufficient to confirm a definitive hemostatic benefit of Magnesium Sulfate in this surgical context.

#### 3.4.3. Opioid Consumption and Analgesic Requirements

**Figure 6:**
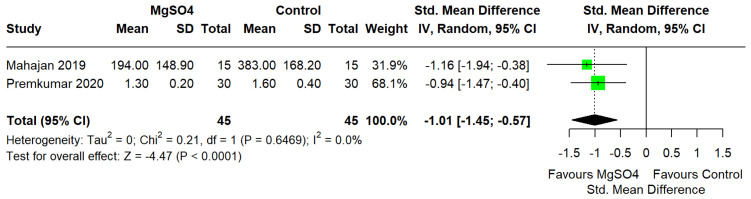
Forest plots of perioperative fentanyl consumption [17,18].

Perioperative fentanyl consumption was analyzed to evaluate the opioid-sparing effect of magnesium sulfate during supratentorial craniotomy. To ensure methodological consistency and address clinical heterogeneity, one study utilizing a regional scalp block administration was excluded, restricting this analysis strictly to intravenous magnesium protocols. Because opioid requirements were reported using different formats across the included studies, the standardized mean difference was selected as the effect measure. The pooled analysis showed that intravenous magnesium sulfate was associated with lower perioperative fentanyl consumption compared with the control group, with a standardized mean difference of −1.01 (95% CI: −1.45 to −0.57; *p* < 0.0001), as presented in Figure 6. Statistical heterogeneity was zero (I^2^ = 0.0%), suggesting a highly consistent opioid-sparing signal across the included trials.

This finding supports the role of magnesium sulfate as an analgesic adjuvant in neurosurgical anesthesia. The observed reduction in fentanyl consumption is biologically plausible because magnesium acts as a non-competitive N-methyl-D-aspartate receptor antagonist and may reduce central sensitization during surgical stimulation. In the context of supratentorial craniotomy, reduced opioid requirement may be clinically relevant because excessive perioperative opioid exposure can delay recovery, increase the risk of respiratory depression, and complicate early neurological assessment. However, this result should be interpreted cautiously because the included trials varied in magnesium dosing protocols, anesthetic maintenance strategies, and perioperative analgesic regimens, and the exclusion of the regional study left only two trials for this pooled analysis, reducing the overall robustness of the conclusion.

#### 3.4.4. Sensitivity Analysis and Heterogeneity Assessment

Heterogeneity was assessed across all outcomes using the I^2^ statistic. The analysis revealed varying degrees of consistency among the included trials. A high degree of heterogeneity was observed in the intraoperative hemodynamic (I^2^ = 73.6%) and blood loss analysis (I^2^ = 92.5%). This variability is likely attributable to clinical diversity, including differences in anesthetic maintenance protocols (TIVA versus inhalational), surgical duration, and measurement techniques across the varying centers. In contrast, the neuroprotective biomarker (I^2^ = 0.0%) and opioid-sparing (I^2^ = 0.0%) outcomes demonstrated zero heterogeneity, indicating consistent physiological responses to intravenous Magnesium Sulfate administration regardless of the specific study setting.

**Figure 7:**
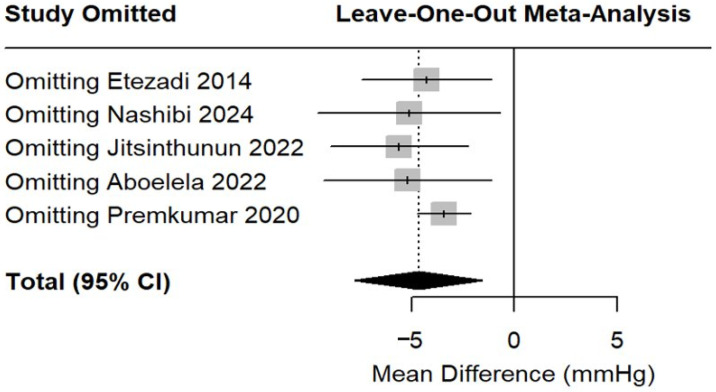
Sensitivity Analysis [15,18,19,20,21].

To explore the robustness of the primary outcome amidst high heterogeneity, a leave-one-out sensitivity analysis was performed for MAP. As depicted in Figure 7, the sequential omission of any single study did not significantly alter the pooled effect estimate, confirming the statistical stability of the findings.

**Figure 8:**
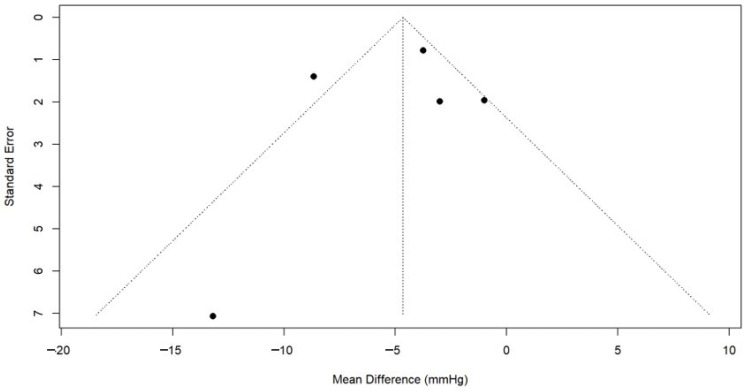
Publication Bias.

Furthermore, despite the limited number of studies included (k < 10), a funnel plot was generated for the primary outcome (MAP) to assess potential reporting bias, in accordance with Cochrane Handbook recommendations. Visual inspection of the funnel plot (Figure 8) reveals slight asymmetry, suggesting the possible presence of small-study effects or publication bias. This limitation must be acknowledged when interpreting the overall effect sizes.

### 3.5. GRADE

**Figure 9:**
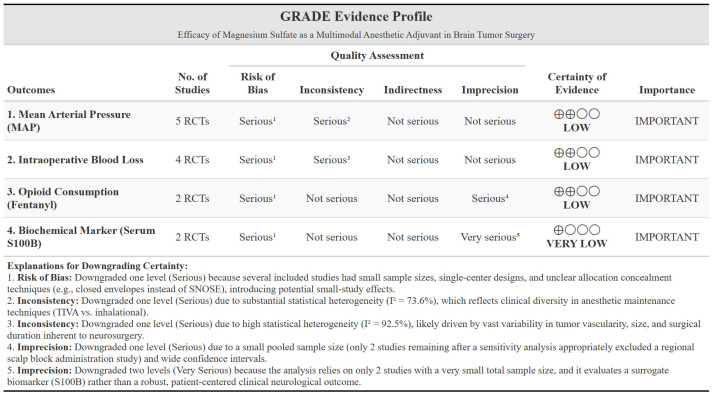
GRADE Evidence Profile.

The certainty of the evidence was systematically evaluated using the GRADE approach following the analysis of all outcomes. The overall quality of evidence ranged from low to very low and was primarily impacted by risk of bias, heterogeneity, and imprecision. As summarized in Figure 8, the evidence for the primary outcome of hemodynamic stability (MAP) was graded as low. This specific outcome was downgraded two levels due to the serious risk of bias (e.g., small sample sizes, single-center designs, unclear allocation concealment) and inconsistency arising from high statistical heterogeneity. Similarly, the certainty for intraoperative blood loss was graded as low owing to a combination of risk of bias and high statistical heterogeneity likely driven by variability in neurosurgical procedures. Following the sensitivity analysis that appropriately excluded a regional administration study, the certainty for opioid consumption was downgraded to low due to risk of bias and serious imprecision resulting from a small pooled sample size. Finally, the outcome related to biochemical evidence of neuroprotection (S100B) was graded as very low because the analysis relies on only two studies with a very small total sample size, and it evaluates a surrogate biomarker rather than a robust, patient-centered clinical neurological outcome (Figure 9).

## 4. Discussion

### 4.1. Hemodynamic Stability and Mechanistic Insights

Our meta-analysis demonstrates that the intraoperative administration of magnesium sulfate significantly attenuates the hemodynamic response to surgical stimuli in supratentorial craniotomy, yielding a pooled mean difference of −4.65 mmHg compared to controls. While statistically significant, the clinical magnitude of this 4.65 mmHg reduction is modest and may not independently translate into major robust postoperative clinical benefits (such as significantly reduced cerebral edema or shorter hospital stays); however, it contributes to an overall smoother intraoperative profile. This finding aligns with, yet distinctively refines, the conclusions of a recent meta-analysis in spinal surgery, which reported that while magnesium acts as a vasodilator, its hypotensive effect is often moderate [7]. In contrast, our analysis in the specific context of craniotomy, a procedure characterized by intense noxious stimuli such as skull pin insertion and dural opening, reveals a statistically significant stabilizing effect. However, it is important to note the substantial heterogeneity observed (I^2^ = 73.6%) in our results, which suggests that while the direction of the effect is beneficial, the magnitude of blood pressure reduction may vary depending on the specific surgical center or patient baseline. Mechanistically, this efficacy is likely attributable to the role of magnesium as a physiological calcium channel blocker. By inhibiting N-type and L-type calcium channels, magnesium dampens the release of norepinephrine from sympathetic nerve terminals and induces direct smooth muscle relaxation [7,23]. This dual mechanism effectively blunts the catecholamine surge associated with noxious neurosurgical stimuli, resulting in a smoother hemodynamic profile rather than precipitating severe hypotension. Furthermore, unlike dexmedetomidine, which has been shown to cause more profound bradycardia in comparative analyses, magnesium sulfate appears to offer a controlled hypotension profile that maintains perfusion pressure within a safe, permissive range (MAP above 60 mmHg) [24,25].

### 4.2. TIVA vs. Inhalational

A critical observation from this study is the persistence of the hemodynamic benefit despite the clinical diversity among the included trials. Although the high overall statistical heterogeneity (I^2^ = 73.6%) reflects variations in study protocols, our exploratory subgroup analysis revealed that heterogeneity was substantially reduced when isolating the Total Intravenous Anesthesia (TIVA) subgroup (I^2^ = 43.5%) compared to the inhalational subgroup (I^2^ = 83.4%). Despite this variance in consistency, the overall pooled estimate remained statistically significant favoring the magnesium group. This suggests that the hemodynamic stabilizing properties of magnesium are robust and potentially independent of the background anesthetic agent, challenging the notion that magnesium might interact less favorably with volatile agents due to their intrinsic vasodilatory properties. While previous studies have primarily focused on magnesium as an adjuvant to general anesthesia broadly, our aggregated data indicates that magnesium acts synergistically with both propofol-based and volatile-based regimens to suppress central sensitization. This has significant clinical implications, as it affords neuroanesthesiologists the flexibility to utilize magnesium sulfate as a standard multimodal adjuvant regardless of their preference for TIVA, which is often favored for motor evoked potential monitoring, or inhalational agents. Consequently, the choice of anesthetic maintenance should be dictated by surgical monitoring requirements, although practitioners should be aware that the magnitude and consistency of magnesium’s hemodynamic effect may be more predictable under TIVA regimens [7,23].

### 4.3. Heterogeneity and Robustness of Evidence

Although the primary hemodynamic analysis presented substantial statistical heterogeneity (I^2^ = 73.6%), this variance likely stems from the inherent clinical diversity across the included trials, specifically differences in baseline surgical invasiveness, anesthetic maintenance protocols, and specific dosing regimens utilized by different centers. Despite this statistical variability, the direction of the treatment effect remained consistent across the majority of studies, consistently favoring the magnesium intervention to reduce arterial pressure. Furthermore, the robustness of this hemodynamic effect was corroborated by our leave-one-out sensitivity analysis, wherein the sequential omission of single studies did not significantly shift the pooled estimate. It is also noteworthy that despite the statistically significant reduction in MAP, the included trials did not report a significant incidence of severe hypotension requiring rescue vasopressors or resulting in new-onset neurological deficits. This supports the safety profile observed in broader meta-analyses, suggesting that when administered with appropriate loading and maintenance doses, magnesium sulfate effectively balances the need for surgical field optimization via controlled hypotension with the imperative of maintaining adequate cerebral perfusion pressure [7,24].

### 4.4. Neuroprotection and Biomarker Analysis

Perhaps the most intriguing finding of this meta-analysis is the demonstration that intraoperative magnesium sulfate significantly reduces postoperative serum S100B levels (SMD = −0.81). Unlike previous reviews that focused solely on hemodynamic parameters, our study provides a quantitative synthesis of this specific biochemical surrogate in the context of supratentorial craniotomy. The analysis revealed a substantial effect size with zero heterogeneity (I^2^ = 0.0%), suggesting a consistent biological response across different clinical settings. S100B is widely recognized as a potential surrogate marker for blood–brain barrier (BBB) disruption and astrocyte injury, and elevated postoperative levels are strongly correlated with the incidence of postoperative cognitive dysfunction (POCD) [26,27]. The observed reduction in S100B levels in the magnesium group is likely mediated by the ability of magnesium to stabilize the neurovascular unit. Experimental models indicate that magnesium preserves endothelial tight junctions and inhibits the apoptotic cascades in neurons by regulating intracellular calcium and reducing oxidative stress [8,28]. Furthermore, by antagonizing NMDA receptors, magnesium prevents the massive influx of calcium and subsequent glutamate excitotoxicity that typically occurs during surgical brain retraction or ischemia. However, it is crucial to acknowledge the limited specificity of S100B, as it can also be released from extracranial sources such as adipose tissue or bone during craniotomy. Therefore, while the reduction in serum S100B observed in our results reflects a favorable biochemical change and implies a potential mitigation of glial injury and BBB leakage, we strictly emphasize that biomarker reduction alone is insufficient to definitively conclude clinically meaningful neuroprotection. These biochemical findings support the hypothesis of magnesium’s protective role but must be validated by future trials utilizing long-term, patient-centered neurological assessments [7,29].

### 4.5. Analgesic Efficacy and Opioid-Sparing Effects

Our results confirm that intravenous magnesium sulfate acts as a potent analgesic adjuvant, evidenced by a statistically significant reduction in perioperative fentanyl consumption (SMD = −1.01) with zero statistical heterogeneity (I^2^ = 0.0%). This lack of heterogeneity indicates that the opioid-sparing effect is highly consistent across the included trials following the specific exclusion of regional administration routes (e.g., scalp blocks), thereby isolating the systemic efficacy of intravenous protocols. This efficacy is pharmacologically explained by the action of magnesium as a non-competitive antagonist at the NMDA receptor site. Surgical trauma triggers the release of excitatory neurotransmitters that activate NMDA receptors, leading to central sensitization, wind-up phenomena, and acute opioid tolerance [28,29]. By blocking these receptors, magnesium prevents the amplification of nociceptive signaling, thereby reducing the total dose of opioids required to achieve adequate analgesia. In the context of neurosurgery, this is particularly advantageous. Reducing the opioid burden directly translates to a lower risk of opioid-induced side effects such as respiratory depression (hypercapnia) and postoperative nausea and vomiting, both of which are critical to avoid as they can precipitously elevate intracranial pressure and complicate neurological recovery [29].

### 4.6. Intraoperative Blood Loss

A common theoretical concern regarding the use of magnesium sulfate is that its vasodilatory properties might impair hemostasis or increase surgical bleeding. However, our meta-analysis dispels this concern, showing that magnesium administration did not significantly increase intraoperative blood loss volume compared to controls. On the contrary, the quantitative synthesis demonstrated a mean reduction of 85.03 mL in the magnesium group, although this difference did not reach statistical significance (*p* = 0.4952). The analysis also revealed high heterogeneity (I^2^ = 92.5%), which likely reflects the vast variability in tumor vascularity, size, and surgical duration inherent to neurosurgery rather than an inconsistent drug effect. The safety profile observed here aligns with findings from spinal surgery meta-analyses, which suggest that magnesium-induced hypotension is distinct from coagulopathy [7]. By facilitating a controlled reduction in mean arterial pressure without compromising platelet function, magnesium may actually improve the surgical field conditions. Therefore, our findings support the conclusion that magnesium sulfate is a safe adjuvant that provides hemodynamic and analgesic benefits without exposing the patient to an increased risk of hemorrhagic complications.

## 5. Conclusions

In conclusion, this systematic review and meta-analysis provides compelling evidence supporting the utility of intravenous magnesium sulfate as a multimodal adjuvant in supratentorial craniotomy. The administration of magnesium was found to significantly attenuate the intraoperative hemodynamic response and reduce perioperative opioid requirements, while also demonstrating biochemical evidence suggestive of a potential neuroprotective benefit through the significant reduction in serum S100B levels. Importantly, regarding safety, the intervention did not significantly alter blood loss volumes compared to controls. However, these findings must be interpreted within the context of certain limitations, including the substantial heterogeneity observed in the hemodynamic and hemostatic outcomes, the variability in dosing regimens and anesthetic maintenance protocols across the included studies, and the relatively small number of trials which warranted a cautious interpretation of the generated funnel plot regarding potential small-study effects and publication bias. Despite these constraints, the consistency of the biomarker and analgesic efficacy suggests that magnesium sulfate contributes positively to physiological stability and should be considered a valuable component of neuroanesthesia protocols, although further large-scale standardized trials with robust clinical neurological endpoints are warranted to refine optimal dosing strategies.

## Figures and Tables

**Table 1:** Summary of Baseline Characteristics and Methodological Features of Included Studies.

Study	Country	Design and Population	Intervention vs. Comparator	Main Results	Strengths and Weaknesses	Primary Outcomes
Etezadi et al., 2014 [15]	Iran	Double-blind RCT N = 60 (Supratentorial Tumor)	Mg: ~214 mg/kg total (pre-emptive Day-2, Day-1, and intra-op) vs. Placebo (Saline)	Propofol requirement and bleeding volume were significantly lower (*p* = 0.002). CRP did not differ.	(+) Pre-emptive prophylactic regimen. (−) Inflammatory parameter is limited to CRP.	MAP, HR, CRP, Bleeding
Mirrahimi et al., 2015 [16]	Iran	Double-blind RCT N = 60 (Supratentorial Tumor)	Mg: ~214 mg/kg total (pre-emptive 3 days) vs. Placebo (Saline)	Significant reduction in S100B (*p* = 0.006), propofol, and bleeding. Barthel Index did not differ.	(+) Correlation of biomarkers with clinical functional outcomes. (−) The 3-day prophylactic regimen is less practical.	S100B, Bleeding, MAP
Mahajan et al., 2019 [17]	India	Double-blind RCT (3-arm) N = 45 (Supratentorial Tumor)	Mg: 50 mg/kg bolus + 25 mg/kg/h vs. Lignocaine and Saline	Post-op VAS and 24 h fentanyl consumption decreased significantly. S-100B decreased only in the Mg group.	(+) Head-to-head comparison with another active agent. (−) Very small sample size per arm (n = 15).	S100B, Fentanyl consumption, VAS
Premkumar et al., 2020 [18]	India	Double-blind RCT N = 60 (Meningioma)	Mg: 30 mg/kg bolus + 10 mg/kg/h vs. Placebo (Saline)	Anesthesia and hemodynamic parameters improved. Post-op seizure incidence did not differ significantly (*p* = 0.27).	(+) Measures serum Mg levels and safety (seizures). (−) Post-operative observation is relatively acute/short.	MAP, HR, Fentanyl requirement
Jitsinthunun et al., 2022 [19]	Thailand	Double-blind RCT N = 76 (Meningioma)	Mg: 40 mg/kg bolus + 10 mg/kg/h vs. Placebo (Saline)	No significant difference in cognitive function (MoCA), bleeding, or anesthesia requirements (*p* > 0.05).	(+) Highly homogeneous population (meningioma only). (−) Negative results, potentially underpowered.	MAP, MoCA score, Bleeding
Aboelela and Alrefaey, 2022 [20]	Egypt	Prospective RCT N = 50 (Supratentorial Tumor)	Mg: 30 mg/kg bolus + 10 mg/kg/h vs. Lidocaine	Significant reduction in isoflurane consumption (*p* = 0.001); faster recovery; stable hemodynamics.	(+) Precise and objective measurement of anesthetic gas. (−) Control uses an active agent rather than a placebo.	MAP, Recovery time, Isoflurane use
Nashibi et al., 2024 [21]	Iran	Double-blind RCT (3-arm) N = 90 (Pituitary Surgery)	Mg: 0.05 mg/kg bolus + 0.015 mg/kg/h vs. Dexmedetomidine and Control	MgSO_4_ is superior to the control regarding the stability of MAP, HR, and bleeding.	(+) Large sample size and comparison with Dexmedetomidine. (−) Different surgical approach (trans-sphenoidal).	MAP, Bleeding, NRS
Gracia et al., 2025 [22]	Spain	Double-blind RCT N = 50 (Supratentorial Tumor)	Mg: ~57 mg/kg bolus + ~285 mg/kg/24 h infusion vs. Placebo (Saline)	Detailed evaluation of S100B, NSE, MRI cavity volume, and neurocognitive function up to 12 months post-op.	(+) Longest follow-up with a comprehensive cognitive test battery. (−) High cumulative dose (24 g).	Brain cavity volume (MRI), S100B
Kadian et al., 2025 [14]	India	Double-blind RCT N = 60 (Craniotomy)	Mg: 50 mg/kg bolus + 15 mg/kg/h vs. Placebo (Saline) (Route: Regional Scalp Block Adjuvant, Not Intravenous)	Requirements for propofol, fentanyl, and sevoflurane were lower; recovery was faster (*p* < 0.001).	(+) Exploration of an innovative regional analgesia route. (−) Route of administration (local/regional) differs from other IV studies.	Fentanyl consumption, CPOT

CPOT, Critical-Care Pain Observation Tool; CRP, C-reactive protein; g, gram; hr, hour; HR, heart rate; intra-op, intraoperative; kg, kilogram; MAP, mean arterial pressure; Mg, magnesium; MgSO_4_, magnesium sulfate; MoCA, Montreal Cognitive Assessment; MRI, magnetic resonance imaging; N, number of participants; NRS, numeric rating scale; NSE, neuron-specific enolase; post-op, postoperative; RCT, randomized controlled trial; S100B, S100 calcium-binding protein B; VAS, visual analogue scale; vs., versus; µg, microgram.

## Data Availability

The original contributions presented in this study are included in the article/Supplementary Materials. Further inquiries can be directed to the corresponding author.

## Supplementary Materials

The following supporting information can be downloaded at https://www.mdpi.com/article/10.3390/jcm15124636/s1. PRISMA 2020 Checklist. Table S1: Detailed search queries and retrieved records by database; Table S2: Domain-Level Risk of Bias Assessment Using the Cochrane RoB 2.0 Tool. References [14,15,16,17,18,19,20,21,22,30] are cited in the Supplementary Materials.

## Abbreviations

The following abbreviations are used in this manuscript:

APSF	Anesthesia Patient Safety Foundation
BBB	Blood–Brain Barrier
BDNF	Brain-Derived Neurotrophic Factor
CI	Confidence Interval
CRP	C-Reactive Protein
GRADE	Grading of Recommendations Assessment, Development, and Evaluation
HR	Heart Rate
ICP	Intracranial Pressure
MAP	Mean Arterial Pressure
MD	Mean Difference
MeSH	Medical Subject Headings
MgSO_4_	Magnesium Sulfate
NMDA	N-Methyl-D-Aspartate
PICOS	Population, Intervention, Comparison, Outcome, Study Design
PONV	Postoperative Nausea and Vomiting
POCD	Postoperative Cognitive Dysfunction
PRISMA	Preferred Reporting Items for Systematic Reviews and Meta-Analyses
PROSPERO	International Prospective Register of Systematic Reviews
RCT	Randomized Controlled Trial
RoB	Risk of Bias
S100B	S100 Calcium-Binding Protein B
SMD	Standardized Mean Difference
TIVA	Total Intravenous Anesthesia

## References

[1] Lizano-Díez I., Poteet S., Burniol-García A., Cerezales M. (2022). The Burden of Perioperative Hypertension/Hypotension: A Systematic Review. PLoS ONE.

[2] Scott M. (2023). Perioperative Patients with Hemodynamic Instability: Consensus Recommendations of the Anesthesia Patient Safety Foundation. Anesth. Analg..

[3] Colombari E., Biancardi V., Colombari D., Katayama P., Medeiros F., Aitken A., Xavier C.H., Pedrino G.R., Bragin D.E. (2025). Hypertension, Blood–brain Barrier Disruption and Changes in Intracranial Pressure. J. Physiol..

[4] Fu Y., Zhou Y., Cui Y., Wu Y., Wang T., Li Y., Yu Y., Han R. (2025). Opioid-Free Anaesthesia and Postoperative Quality of Recovery in Patients Undergoing Supratentorial Tumour Resection: Protocol for A Randomised Controlled Trial. BMJ Open.

[5] Salomé A., Harkouk H., Fletcher D., Martinez V. (2021). Opioid-Free Anesthesia Benefit–Risk Balance: A Systematic Review and Meta-Analysis of Randomized Controlled Trials. J. Clin. Med..

[6] Song B., Li W., Wan L., Zhang L. (2025). Effect of Opioid-Free Versus Opioid Anesthesia on the Quality of Postoperative Recovery in Patients Receiving Laparoscopic Sleeve Gastrectomy. Obes. Surg..

[7] Campos J., Bas J., Campos C., Mariscal G., Bas T., Bas P. (2024). Efficacy and Safety of Intravenous Magnesium Sulfate in Spinal Surgery: A Systematic Review and Meta-Analysis. J. Clin. Med..

[8] Cazzaniga A., Fedele G., Castiglioni S., Maier J. (2022). The Presence of Blood–Brain Barrier Modulates the Response to Magnesium Salts in Human Brain Organoids. Int. J. Mol. Sci..

[9] Masri A., Corell A., Michaëlsson I., Jakola A., Skoglund T. (2024). The Glymphatic System for Neurosurgeons: A Scoping Review. Neurosurg. Rev..

[10] Kulik K., Żyżyńska-Granica B., Kowalczyk A., Kurowski P., Gajewska M., Bujalska-Zadrożny M. (2021). Magnesium and Morphine in the Treatment of Chronic Neuropathic Pain–A Biomedical Mechanism of Action. Int. J. Mol. Sci..

[11] Sohn H., Kim B., Bae Y., Seo W., Jeon Y. (2021). Magnesium Sulfate Enables Patient Immobilization during Moderate Block and Ameliorates the Pain and Analgesic Requirements in Spine Surgery. J. Clin. Med..

[12] Myers A., Baker D., Adedugbe I. (2023). Magnesium, the Anaesthetists’ Friend during Carotid Endarterectomy (CEA). BJS.

[13] Fiorentini D., Cappadone C., Farruggia G., Prata C. (2021). Magnesium: Biochemistry, Nutrition, Detection, and Social Impact of Diseases Linked to Its Deficiency. Nutrients.

[14] Kadian S., Gupta P., Agrawal S. (2025). Comparative Evaluation of Analgesic Efficacy of Ketamine and Magnesium Sulfate as Adjuvants to Bupivacaine for Scalp Block in Supratentorial Craniotomy: A Randomized, Double-Blind Clinical Study. J. Clin. Neurosci..

[15] Etezadi F., Aklamli M., Najafi A., Khajavi M., Moharari R.S., Mirrahimi B., Mortazavi S.A., Mojtahedzadeh M. (2014). Evaluation of the Anti-Inflammatory Effects of Peri-Operative Infusion of Magnesium Sulfate on the Microsurgical Procedures for Intracranial Tumors. Anesthesiol. Pain Med..

[16] Mirrahimi B., Mortazavi A., Nouri M., Ketabchi E., Amirjamshidi A., Ashouri A., Khajavi M., Mojtahedzadeh M. (2015). Effect of Magnesium on Functional Outcome and Paraclinical Parameters of Patients Undergoing Supratentorial Craniotomy for Brain Tumors: A Randomized Controlled Trial. Acta Neurochir..

[17] Mahajan C., Mishra R.K., Jena B.R., Kapoor I., Prabhakar H., Rath G.P., Chaturvedi A. (2019). Effect of Magnesium and Lignocaine on Post-Craniotomy Pain: A Comparative, Randomized, Double Blind, Placebo-Controlled Study. Saudi J. Anaesth..

[18] Premkumar D., Dhawale Y., Agarwal A. (2020). Effect of Intravenous Magnesium Sulfate on Patients Undergoing Craniotomy for Meningioma Excision. Glob. J. Res. Anal..

[19] Jitsinthunun T., Raksakietisak M., Pantubtim C., Mahatnirunkul P. (2022). Effects of Magnesium Sulfate on Intraoperative Blood Loss and Anesthetic Requirement in Meningioma Patients Undergoing Craniotomy with Tumor Removal: A Prospective Randomized Study. J. Neuroanaesth. Crit. Care.

[20] Aboelela M.A., Alrefaey A.K. (2022). Lidocaine versus Magnesium Sulfate Infusion during Isoflurane Anesthesia for Brain Tumor Resection, Effect on Minimum Alveolar Concentration Reduction Guided by Bispectral Index: A Prospective Randomized Controlled Trial. Signa Vitae.

[21] Nashibi M., Sezari P., Nashibi S., Pilevar N., Safari F., Hosseininasab S.-S.-M., Asgari S. (2024). A Comparison of Dexmedetomidine and Magnesium Sulfate as an Anesthetic Adjuvant in Trans-Sphenoidal Pituitary Surgery. J. Cell. Mol. Anesth..

[22] Gracia I., Fabregas N., Hurtado P., De Riva N., Boget T., Casanovas G., Oleaga L., Bargalló N., González J., Rumià J. (2025). Effect of Perioperative Magnesium Sulfate on Neurological Outcome in Neurosurgical Patients: A Randomized Double-Blind Controlled Trial. Minerva Anestesiol..

[23] Sohn H., Ahn H., Seo W., Yi I., Park J. (2022). Magnesium Sulfate and Cerebral Oxygen Saturation in Mild Traumatic Brain Injury: A Randomized, Double-Blind, Controlled Trial. J. Clin. Med..

[24] Lang B., Zhang L., Lin Y., Zhang W., Li F., Chen S. (2020). Comparison of Effects and Safety in Providing Controlled Hypotension during Surgery between Dexmedetomidine and Magnesium Sulphate: A Meta-Analysis of Randomized Controlled Trials. PLoS ONE.

[25] D’Amico F., Fominskiy E., Turi S., Pruna A., Fresilli S., Triulzi M., Zangrillo A., Landoni G. (2023). Intraoperative Hypotension and Postoperative Outcomes: A Meta-Analysis of Randomised Trials. Br. J. Anaesth..

[26] Gan L., Qian K., Yang J., Cai Q., Ye Q., Dai M., Jia Z., Jiang T., Ma C., Lin X. (2025). Intraoperative Transcutaneous Electrical Acupoint Stimulation Combined with Anesthesia to Prevent Postoperative Cognitive Dysfunction: A Systematic Review and Meta-Analysis. PLoS ONE.

[27] Wang X., Chen X., Wu F., Liu Y., Yang Y., Chen W., Pan Z., Hu W., Zheng F., He H. (2023). Relationship between Postoperative Biomarkers of Neuronal Injury and Postoperative Cognitive Dysfunction: A Meta-Analysis. PLoS ONE.

[28] Zhang Y., Jiang M., Wei M., Wu C., Huang Y., Song B., Xu Y., Zhang H., Shen Y., Wu D. (2025). MgSO_4_ as a Novel Hypothermia Infusion Solution Promotes Ischemic Stroke Recovery through Ca^2+^ Regulation of Neurovascular Units. Theranostics.

[29] Lee Y., Kim B., Park J., Kim S., Park H. (2020). The Effect of Intraoperative Magnesium Sulphate Infusion on Emergence Agitation after Ambulatory Ophthalmic Surgery in Children. J. Clin. Med..

[30] Page M.J., McKenzie J.E., Bossuyt P.M., Boutron I., Hoffmann T.C., Mulrow C.D., Shamseer L., Tetzlaff J.M., Akl E.A., Brennan S.E. (2021). The PRISMA 2020 statement: An updated guideline for reporting systematic reviews. BMJ.

