# Supplementary material for: Magnesium Sulfate as a Multimodal Anesthetic Adjuvant in Brain Tumor Surgery: A Systematic Review and Meta-Analysis of Hemodynamic, Analgesic, and Biomarker Outcomes
Source: J Clin Med. 2026 Jun 15;15(12):4636. doi: 10.3390/jcm15124636 (PMC13301512; doi:10.3390/jcm15124636)
Supplement: Supplementary file 1 [file jcm-15-04636-s001.zip › Suppelementary Table.pdf]

Supplementary

Supplementary Table S1. Detailed search queries and retrieved records by database.

Database	Search Strategy			Hits
Pubmed	#1	("Magnesium Sulfate"[MeSH Terms] OR "magnesium sulfate"[tiab] OR "MgSO4"[tiab] OR Magnesium [tiab])	#1 AND #2 AND #3	37
	#2	("Craniotomy"[MeSH Terms] OR "craniotomy"[tiab] OR "neurosurgery"[tiab])		
	#3	(brain neoplasm [MeSH Terms] OR "brain tumor"[tiab] OR "brain neoplasm"[tiab] OR "supratentorial"[tiab] OR "meningioma"[tiab])		
Scopus		Magnesium AND craniotomy AND "brain tumor" AND (neuroprotect* OR hemodynamic OR analges* OR "cognitive function")		9
EBSCO		(MH "Magnesium Sulfate" OR TX "magnesium sulfate" OR TX "MgSO4") AND (MH "Craniotomy" OR TX "craniotomy" OR TX "neurosurgery") AND (MH "Brain Neoplasms" OR TX "brain tumor" OR TX "brain neoplasm" OR TX "supratentorial" OR TX "meningioma") AND (MH "Neuroprotection" OR TX "neuroprotective" OR TX "S100B" OR TX "NSE" OR TX "MoCA" OR TX "Barthel Index" OR TX "CRP" OR TX "IL-6" OR TX "TNF-alpha" OR TX "hemodynamic" OR TX "MAP" OR TX "HR" OR TX "anesthetic requirement" OR TX "propofol" OR TX "opioid" OR TX "seizure") AND (TX "randomized" OR TX "randomized controlled trial" OR TX "RCT")		26
Cochrane CENTRAL	ti,ab,kw	(magnesium sulfate OR MgSO4) AND (craniotomy OR neurosurgery) AND (brain tumor OR brain neoplasm OR supratentorial OR meningioma) AND (neuroprotection OR neuroprotective OR S100B OR NSE OR MoCA OR "Barthel Index" OR CRP OR IL-6 OR "TNF-alpha" OR hemodynamic OR MAP OR HR OR "anesthetic requirement*" OR propofol OR opioid OR seizure)		16

Supplementary Table S2. Domain-Level Risk of Bias Assessment Using the Cochrane RoB 2.0 Tool.

**Supplementary Table S2.** Domain-Level Risk of Bias Assessment Using the Cochrane RoB 2.0 Tool						
**Study**	**D1: Randomization Process**	**D2: Deviations from Intended Interventions**	**D3: Missing Outcome Data**	**D4: Measurement of the Outcome**	**D5: Selection of the Reported Result**	**Overall Bias**
**Premkumar 2020**	**Low**	**Low**	**Low**	**Low**	**Some concerns**	**Some concerns**
	Adequate randomization with computer-generated sequence. Allocation concealment achieved via sealed opaque envelopes.	Double-blind design with identical placebo preparation. No relevant protocol deviations reported.	Complete outcome data for all 60 randomized participants. No missing data or dropout.	Standardized hemodynamic monitoring (MAP, HR) and perioperative drug use recorded objectively.	No pre-registered analysis plan identified (PROSPERO or ClinicalTrials.gov). Outcome selection transparency limited.	Some concerns: one or more domains with 'Some concerns,' primarily D1 or D5.
**Nashibi 2024**	**Low**	**Low**	**Low**	**Low**	**Some concerns**	**Some concerns**
	Computer-generated randomization in this three-arm RCT (n=90). Allocation concealment maintained until assignment.	Double-blind administration with standardized drug preparation across all three arms. No deviations reported.	All enrolled participants included in final analysis; complete follow-up for all primary and secondary endpoints.	Objective hemodynamic and pain score measurements. Outcome assessors not involved in drug administration.	No publicly available pre-specified statistical analysis plan. Risk of selective outcome reporting cannot be fully excluded.	Some concerns: one or more domains with 'Some concerns,' primarily D1 or D5.
**Kadian 2025**	**Low**	**Low**	**Low**	**Low**	**Low**	**Low**

**Supplementary Table S2.** Domain-Level Risk of Bias Assessment Using the Cochrane RoB 2.0 Tool						
**Study**	**D1: Randomization Process**	**D2: Deviations from Intended Interventions**	**D3: Missing Outcome Data**	**D4: Measurement of the Outcome**	**D5: Selection of the Reported Result**	**Overall Bias**
	Computer-generated random number sequence with concealed allocation. Prospectively registered trial.	Double-blind; MgSO4 and ketamine prepared identically by pharmacist. No protocol deviations identified.	All 60 randomized participants completed the study with no missing outcome data.	Validated pain assessment tools (CPOT, NRS) and objective drug consumption measurements used throughout.	Study prospectively registered; pre-specified outcomes aligned with reported results. No selective reporting concern.	Low: all domains rated low risk.
	**Low**	**Low**	**Low**	**Low**	**Some concerns**	**Some concerns**
**Gracia 2025**	Block randomization with adequate sealed envelope allocation concealment; baseline characteristics balanced.	Double-blind placebo-controlled design. No relevant deviations from intended interventions described.	Complete long-term follow-up including 12-month neurocognitive assessments in all participants.	Validated neurocognitive battery, blinded MRI evaluation, and centralized biomarker (S100B, NSE) assay used.	Published trial protocol not publicly available. Some uncertainty regarding pre-specification of all secondary endpoints.	Some concerns: one or more domains with 'Some concerns,' primarily D1 or D5.
	**Some concerns**	**Some concerns**	**Low**	**Low**	**Some concerns**	**Some concerns**
**Mahajan 2019**	Randomization method not clearly described. Allocation concealment	Three-arm design comparing MgSO4, lignocaine, and saline; limited blinding detail for	All 45 participants' outcome data available for analysis. No dropout or	VAS assessed by blinded evaluator; S100B measured in standardized laboratory.	No prospectively registered protocol identified. Reporting limited to pre-selected time	Some concerns: one or more domains with 'Some concerns,' primarily D1 or D5.

Supplementary Table S2. Domain-Level Risk of Bias Assessment Using the Cochrane RoB 2.0 Tool

Study	D1: Randomization Process	D2: Deviations from Intended Interventions	D3: Missing Outcome Data	D4: Measurement of the Outcome	D5: Selection of the Reported Result	Overall Bias
	procedure not adequately reported (NI). Small sample size per arm (n=15) raises concern for imbalance.	drug preparation staff. Partial awareness of intervention assignment possible.	protocol withdrawal documented.	Objective pain and biomarker metrics.	points; selective reporting cannot be fully excluded.	
Mirrahimi 2015	**Some concerns**	**Low**	**Low**	**Low**	**Some concerns**	**Some concerns**
	Randomization process not fully described. Allocation concealment method not reported (NI). Three-day preoperative regimen raises practical concealment concerns.	Double-blind placebo-controlled design. No deviations from intended three-day preoperative MgSO4 protocol reported.	Complete outcome data for all 60 randomized participants. Barthel Index and biomarker data fully available.	Standardized S100B assay and objective hemodynamic monitoring. Outcome assessment blinded to group allocation.	No pre-registered protocol identified. Functional outcome (Barthel Index) reporting appears complete but analysis plan transparency is limited.	Some concerns: one or more domains with 'Some concerns,' primarily D1 or D5.
Aboelela 2021	**High**	**Low**	**Low**	**Low**	**Some concerns**	**High**
	Randomization method not	Single-blinded design; however,	Complete outcome data for all 50	Bispectral index (BIS) monitoring	No pre-registered analysis plan	High: driven by D1 (inadequate randomization/concealment).

Supplementary Table S2. Domain-Level Risk of Bias Assessment Using the Cochrane RoB 2.0 Tool

Study	D1: Randomization Process	D2: Deviations from Intended Interventions	D3: Missing Outcome Data	D4: Measurement of the Outcome	D5: Selection of the Reported Result	Overall Bias
	described. No allocation concealment procedure reported. Absence of adequate randomization significantly elevates risk of selection bias (High risk).	no relevant deviations from the intended intervention (MgSO4 vs. lidocaine) were identified during the study period.	participants. No participant dropout or missing endpoint data reported.	and objective isoflurane gas measurement provide standardized, objective outcome assessment.	identified. Active comparator (lidocaine) design introduces potential selective reporting of comparative outcomes.	
Etezadi 2014	Low	Low	Low	Low	Some concerns	Some concerns
	Adequate randomization with sealed envelope allocation concealment. Balanced baseline characteristics across groups.	Double-blind design with identical placebo preparation. No protocol deviations or co-intervention imbalances reported.	All enrolled participants included in final analysis. No missing data for primary or secondary outcomes.	Standardized intraoperative MAP monitoring and CRP laboratory measurement. Objective, blinded outcome assessment.	No prospective trial registration identified. Pre-specified analysis plan not publicly available; selective reporting risk cannot be excluded.	Some concerns: one or more domains with 'Some concerns,' primarily D1 or D5.
Jitsinthunun 2022	Low	Low	Low	Low	Some concerns	Some concerns

**Supplementary Table S2.** Domain-Level Risk of Bias Assessment Using the Cochrane RoB 2.0 Tool						
**Study**	**D1: Randomization Process**	**D2: Deviations from Intended Interventions**	**D3: Missing Outcome Data**	**D4: Measurement of the Outcome**	**D5: Selection of the Reported Result**	**Overall Bias**
	Computer-generated random number sequence with concealed allocation. Homogeneous meningioma population.	Double-blind placebo-controlled design. Standard blinding procedures maintained throughout. No protocol deviations.	Complete intention-to-treat analysis for all 76 randomized participants. No dropout or missing data.	Validated MoCA cognitive tool and standardized intraoperative monitoring. Blinded outcome assessment for cognitive endpoints.	No prospective protocol registration identified. Multiple secondary outcome measurements create potential for selective reporting.	Some concerns: one or more domains with 'Some concerns,' primarily D1 or D5.
**Legend:** D1 = Bias arising from the randomization process; D2 = Bias due to deviations from intended interventions (ITT effect); D3 = Bias due to missing outcome data; D4 = Bias in measurement of the outcome; D5 = Bias in selection of the reported result. **Color coding:** Low risk (green), Some concerns (amber), High risk (red). RoB 2.0 = Cochrane Risk of Bias tool version 2.0; NI = No information; ITT = Intention-to-treat.						

Abbreviations: D1-D5: RoB 2.0 domains; NI: No information; ITT: Intention-to-treat; MAP: Mean Arterial Pressure; MgSO4: Magnesium Sulfate; RCT: Randomized Controlled Trial; VAS: Visual Analogue Scale; MoCA: Montreal Cognitive Assessment; CPOT: Critical-Care Pain Observation Tool; BIS: Bispectral Index; SAP: Statistical Analysis Plan; CRP: C-Reactive Protein; NSE: Neuron-Specific Enolase; S100B: S100 Calcium-Binding Protein B.
